# A Polymeric Two‐in‐One Electron Transport Layer and Transparent Electrode for Efficient Indoor All‐Organic Solar Cells

**DOI:** 10.1002/advs.202405676

**Published:** 2024-08-29

**Authors:** Tiefeng Liu, Gulzada Beket, Qifan Li, Qilun Zhang, Sang Young Jeong, Chi‐Yuan Yang, Jun‐Da Huang, Yuxuan Li, Marc‐Antoine Stoeckel, Miao Xiong, Tom P. A. van der Pol, Jonas Bergqvist, Han Young Woo, Feng Gao, Mats Fahlman, Thomas Österberg, Simone Fabiano

**Affiliations:** ^1^ Laboratory of Organic Electronics Department of Science and Technology Linköping University Norrköping SE‐60174 Sweden; ^2^ Wallenberg Initiative Materials Science for Sustainability Department of Science and Technology Linköping University Norrköping SE‐60174 Sweden; ^3^ Electronic and Photonic Materials Department of Physics Chemistry, and Biology Linköping University Linköping SE‐58183 Sweden; ^4^ Epishine AB Attorpsgatan 2 Linköping SE‐58273 Sweden; ^5^ Wallenberg Wood Science Center Department of Science and Technology (ITN) Linköping University Norrköping SE‐60174 Sweden; ^6^ Department of Chemistry College of Science Korea University 145 Anam‐ro, Seongbuk‐gu Seoul 02841 Republic of Korea; ^7^ n‐Ink AB Bredgatan 33 Norrköping SE‐60174 Sweden

**Keywords:** conducting polymers, indoor all‐organic solar cell, low work function electrode, PBFDO, PEDOT:PSS

## Abstract

Transparent electrodes (TEs) are vital in optoelectronic devices, enabling the interaction of light and charges. While indium tin oxide (ITO) has traditionally served as a benchmark TE, its high cost prompts the exploration of alternatives to optimize electrode characteristics and improve device efficiencies. Conducting polymers, which combine polymer advantages with metal‐like conductivity, emerge as a promising solution for TEs. This work introduces a two‐in‐one electron transport layer (ETL) and TE based on films of polyethylenimine ethoxylated (PEIE)‐modified poly(benzodifurandione) (PBFDO). These PEIE‐modified PBFDO layers exhibit a unique combination of properties, including low sheet resistance (130 Ω sq^−1^), low work function (4.2 eV), and high optical transparency (>85% in the UV–vis‐NIR range). In contrast to commonly used poly(3,4‐ethylenedioxythiophene):poly(styrenesulfonate) (PEDOT:PSS), the doping level of PBFDO remains unaffected by the PEIE treatment, as verified through UV–vis‐NIR absorption and X‐ray photoelectron spectroscopy measurements. When employed as a two‐in‐one ETL/TE in organic solar cells, the PEIE‐modified PBFDO electrode exhibits performance comparable to conventional ITO electrodes. Moreover, this work demonstrates all‐organic solar cells with record‐high power conversion efficiencies of >15.1% under indoor lighting conditions. These findings hold promise for the development of fully printed, all‐organic optoelectronic devices.

## Graphical abstract

Transparent electrodes are crucial in optoelectronic devices. This study presents a two‐in‐one electron transport layer and transparent electrodes using polyethylenimine ethoxylated (PEIE)‐modified poly(benzodifurandione) (PBFDO) films, offering low resistance, low work function, and high transparency. These layers achieve performance comparable to ITO electrodes, enabling all‐organic solar cells with >15.1% efficiency under indoor lighting, promising for fully printed devices.

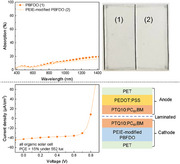

## 1 Introduction

Transparent electrodes (TEs) play a crucial role in optoelectronic devices, facilitating the in‐ and out‐coupling of light and charges.^[^ ^1^ ^]^ Indium tin oxide (ITO) stands out as a benchmark TE in both academic research and industrial applications, owing to its exceptional conductivity and transmittance.^[^ ^2^ ^]^ Despite its effectiveness, the increasing demand and cost associated with the rare element indium motivate the search for alternative TEs with optimized electrode characteristics and enhanced device efficiencies.^[^ ^3^ ^]^

Various metal oxides, such as zinc oxide (ZnO)^[^ ^4^ ^]^ and tin oxide (SnO_2_)^[^ ^5^ ^]^ have been explored as partial substitutes for ITO electrodes in specific applications. However, these metal‐oxide electrodes face limitations for the development of flexible devices due to their mechanical fragility and the requirement for a high‐temperature sintering process. In response to these challenges, metallic nanostructures like metal grids^[^ ^6^ ^]^ and metal nanowires,^[^ ^7^ ^]^ as well as carbon‐based materials such as carbon nanotubes^[^ ^8^ ^]^ and graphene,^[^ ^9^ ^]^ have emerged as promising alternatives. These materials enable the fabrication of thin and flexible films with low sheet resistance and high transparency. However, their suitability for large‐scale applications is hindered by the high surface roughness and intricate synthetic routes.^[^ ^10^ ^]^

**Figure 1:**
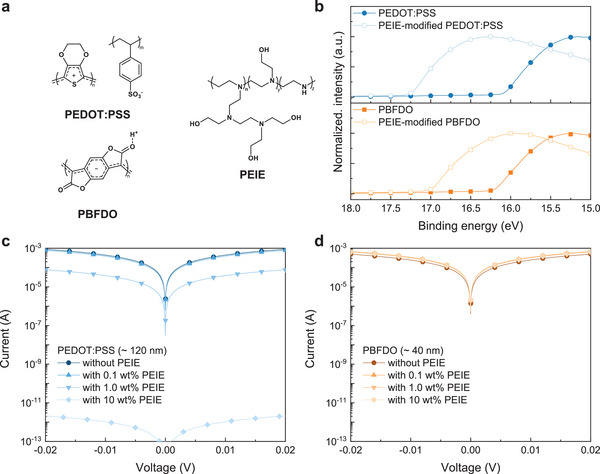
a) Chemical structures of PEDOT:PSS, PBFDO, and PEIE. b) Secondary electron cutoff spectra of PEDOT:PSS and PBFDO films before and after PEIE treatment. Current‐voltage characteristics of c) PEDOT:PSS and d) PBFDO films modified by PEIE solution at different concentrations.

Conducting polymers are highly promising materials for TEs, combining the advantageous features of both polymers (such as solution processability, mechanical flexibility, lightweight, and chemical versatility) and metals (electrical conductivity).^[^ ^11^ ^]^ Although several conducting polymers can achieve electrical conductivity exceeding 1000 S cm^−1^, comparable to that of ITO, their usage as TEs has been typically restricted by a lack of stability at high doping levels.^[^ ^12^ ^]^ The p‐type poly(3,4‐ethylenedioxythiophene):poly(styrenesulfonate) (PEDOT:PSS, **Figure** **1**a) stands out for its remarkable ambient and operational stability and solution processibility even at high doping levels. It finds wide applications in various optoelectronic devices, such as organic solar cells and light‐emitting diodes,^[^ ^13^ ^]^ primarily serving as a high work function (WF) TE to extract or inject holes from the active layer due to its suitable WF.^[^ ^14^ ^]^ When a PEDOT:PSS electrode is used to inject or extract electrons, an additional low‐WF modifier or interlayer is needed to reduce its WF.^[^ ^15^ ^]^ However, these modifiers or interlayers often contain amine groups, such as poly(ethyleneimine) (PEI), polyethylenimine ethoxylated (PEIE), and ethanolamine‐based sol‐gel ZnO precursors, leading to a chemical reduction of PEDOT:PSS (i.e., reduced doping level) during the modifier/interlayer deposition process.^[^ ^16^ ^]^ This de‐doping process increases its sheet resistance and decreases transparency in the visible region, resulting in poor device performance.^[^ ^17^ ^]^ While various strategies, such as passivation of amine by metal chelation^[^ ^18^ ^]^ and novel precursor engineering,^[^ ^19^ ^]^ can mitigate this undesirable interface reaction, there is still a pressing need to explore chemically stable conducting polymers to act as low‐WF electrodes.

Intuitively, the use of electron‐transporting (n‐type) organic conductors could address this issue. It has been shown, for example, that treating n‐type semiconductors such as poly{[N,N′‐bis(2‐octyldodecyl)naphthalene‐1,4,5,8‐bis(dicarboximide)‐2,6‐diyl]‐alt‐5,5′‐(2,2′‐bithiophene)} [P(NDI2OD‐T2)], poly(benzimidazobenzophenanthroline) (BBL), or [6,6]‐phenyl‐C_61_‐butyric acid methyl ester (PC_61_BM) with amine‐based polymers like PEI or PEIE yields highly conductive, low‐WF TEs.^[^ ^20^ ^]^ However, the inevitable electron transfer between these amine‐based modifiers/interlayers and the n‐type semiconductors also leads to a chemical reduction of the latter (i.e., increased doping level), which forces the n‐type semiconductors out of their thermodynamic equilibrium, thus affecting their ambient stability. We hypothesize that the use of amine‐based modifiers on intrinsically doped n‐type conducting polymers, such as the recently reported poly(3,7‐dihydrobenzo[1,2‐b:4,5‐b′]difuran‐2,6‐dione) (PBFDO),^[^ ^21^ ^]^ would not alter their charge carrier density, and thus intrinsic electrical conductivity and optical properties, but would instead only influences their energetics. This would then enable the development of a two‐in‐one electron transport layer (ETL) and TE with ideal and stable electrical, optical, and electronic properties.

Here, we report a two‐in‐one ETL/TE characterized by high electrical conductivity, high optical transparency in the visible range, and low WF. We demonstrate that spin‐coating a thin film of PEIE on top of the n‐type PBFDO (Figure 1a) layer yields conductive films having a unique combination of low sheet resistance (130 Ω sq^−1^), low WF (4.2 eV), and high transparency (>85%) in the UV–vis‐NIR range. Unlike the commonly used PEDOT:PSS and other n‐type semiconductors, the doping level of PBFDO is not affected by the deposition of the amine‐based PEIE, as confirmed by a combination of UV–vis‐NIR absorption and X‐ray photoelectron spectroscopy (XPS) measurements. When used as the two‐in‐one ETL/TE in OSCs, the PEIE‐modified PBFDO electrode demonstrates comparable performance to the commonly used ITO electrode. Furthermore, through a straightforward lamination technique, we successfully fabricated all‐organic solar cells with a record‐high power conversion efficiency (PCE) of > 15.1% under indoor lighting conditions. This result holds promising implications for the realization of fully printed solar cells.

## 2 Results and Discussion

PBFDO has recently emerged as a notable example of n‐type conducting polymers, boasting an impressive electrical conductivity that exceeds 2000 S cm^−1^, comparable to commercial p‐type PEDOT:PSS formulations.^[^ ^21^, ^22^ ^]^ Similar to PEDOT:PSS, PBFDO is transparent in the visible spectrum and possesses a high WF exceeding 5 eV, rendering it stable in ambient conditions. However, while this high WF is advantageous for stability,^[^ ^23^ ^]^ it diminishes its efficiency in extracting electrons from commonly used OSC materials, hampering its application as ETL.

To address this limitation, we deposited an ultrathin layer (< 10 nm) of PEIE on top of PBFDO to induce a shift of the WF toward lower energies. This shift was confirmed through ultraviolet photoemission spectroscopy (UPS, Figure 1b) and Kelvin probe measurements conducted in ambient conditions (Figure S1, Supporting Information), which indicated a transition from 5.03 eV for pristine PBFDO to 4.21 eV for PEIE‐modified PBFDO. For comparison, the application of an ultrathin layer of PEIE on PEDOT:PSS yields a similar WF shift, with the WF changing from 5.14 eV for pristine PEDOT:PSS to 3.97 eV for PEIE‐modified PEDOT:PSS (Figure 1b). A similar trend is observed when coating commonly used sol‐gel ethanolamine‐based ZnO precursors containing ethanolamine on top of PEDOT:PSS and PBFDO films, with their WF shifting from ≈5.0 to ≈3.9 eV for both polymers upon treatment (Figure S2a,b, Supporting Information).

In stark contrast to PEDOT:PSS, the application of PEIE on top of PBFDO does not induce any change in sheet resistance, which instead remains relatively constant at ≈138.7 ± 5.5 Ω sq^−1^ regardless of the PEIE concentration (i.e., PEIE film thickness), as shown in Figure 1c,d and Figure S3, Supporting Information. Similarly, applying the sol‐gel ethanolamine‐based ZnO precursors to coat both PEDOT:PSS and PBFDO films induces a 10× increase in the sheet resistance of PEDOT:PSS, while that of PBFDO remains constant (Figure S2c,d, Supporting Information).

**Figure 2:**
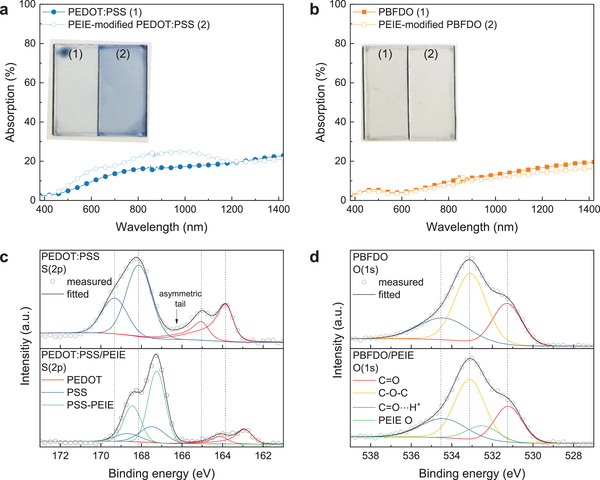
Absorption spectra of a) PEDOT:PSS and b) PBFDO films before and after treatment with 1 wt% PEIE solution. The absorption was obtained from the transmittance and reflectance reported in Figure S4, Supporting Information. Photographs of the corresponding films are shown in the insets. c) S(2p) XPS spectra of PEDOT films and d) O(1s) XPS spectra of PBFDO films before and after modification with PEIE.

Since the electrical properties of conducting polymers are intricately tied to their optical characteristics, we measured the absorption properties of PEDOT:PSS and PBFDO before and after PEIE treatment to investigate the effect of PEIE on their doping level. As illustrated in **Figure** **2**a, the absorption spectrum of pristine PEDOT:PSS films features a broad absorption band extending far into the infrared region, attributed to polaron/bipolaron absorptions.^[^ ^24^ ^]^ Upon depositing PEIE on PEDOT:PSS, the absorption band in the region 500–1100 nm increases while the extended absorption band at wavelengths larger than 1200 nm decreases. The alteration is visually apparent in the optical photograph reported as an inset to Figure 2a, where the color of the PEDOT:PSS films transitions from light blue to dark blue, providing clear evidence of dedoping.^[^ ^20c^ ^]^ Conversely, PBFDO films exhibit negligible changes in absorption and color after PEIE modification (Figure 2b), suggesting that the doping level of PBFDO remains largely unaltered. The corresponding transmittance and reflectance spectra are reported in Figure S4, Supporting Information for a detailed analysis.

Next, we conducted X‐ray photoelectron spectroscopy (XPS) studies to understand better how the PEIE treatment affects the reduction/oxidation state of PBFDO and PEDOT:PSS films. As shown in Figure 2c, the S(2p) signal in the pristine PEDOT:PSS film can be divided into two components. The first at lower binding energy (167–163 eV) originates from the thiophene group in the PEDOT chain, while the second at higher binding energy (171–167 eV) arises from the sulphonate group in the PSS chain.^[^ ^25^ ^]^ Deconvolution of the PEDOT component reveals a strong asymmetric doublet, characterized by a tail extending toward higher binding energies. This asymmetry is indicative of the presence of positive charges delocalized over the PEDOT chains, resulting in a change in the electronic environment near the sulfur atom. Following PEIE modification, the signal of the PEDOT component decreases compared to that of the PSS component, and the asymmetric tail becomes weaker. These changes suggest a significant decrease in PEDOT's oxidation (doping) level upon PEIE treatment.^[^ ^20c^ ^]^ The N(1s) signal, presented in Figure S5, Supporting Information, exhibits a prominent peak at ≈402.3 eV, attributed to positively charged amines formed during the de‐doping process.^[^ ^26^ ^]^ The de‐doping of PEDOT:PSS is further confirmed by electron paramagnetic resonance (EPR) spectroscopy measurements (Figure S6, Supporting Information), showing a gradual decrease of the EPR signal with increasing PEIE concentration.

For PBFDO, the O(1s) signal originates from the lactone group. In the n‐doped PBFDO, protons are expected to compensate for the negative charges on the polymer backbone, causing the O(1s) signal from the carbonyl group to shift toward higher binding energies.^[^ ^21^ ^]^ The reduction level of PBFDO can be evaluated by assessing the peak area of the protonated carbonyl group at 534.5 eV and the pristine carbonyl group at 531.3 eV. As shown in Figure 2d, the signal ratio of two carbonyl groups remains nearly unchanged after PEIE modification, indicating that the reduction level of PBFDO film does not change with the treatment. The EPR signal of PBFDO films is not affected by the PEIE treatment and remains independent of the PEIE concentration (Figure S6, Supporting Information).

We also examined the morphology and microstructure of PEIE‐modified PBFDO films. Atomic force microscopy (AFM) images, presented in Figure S7, Supporting Information, reveal that both PBFDO and PEIE‐modified PBFDO films exhibit a smooth and uniform surface, which is advantageous for the fabrication of multi‐layer thin‐film devices. Subjecting PEIE‐modified PBFDO deposited on a flexible PET substrate to over 1000 bending cycles (radius 2.5 mm) does not cause significant changes in morphology or the appearance of cracks in the film (Figure S8, Supporting Information). This is consistent with the PEIE‐modified PBFDO film retaining over 90% of its electrical conductivity even after 1000 bending cycles (Figure S9, Supporting Information). Grazing‐incidence wide‐angle X‐ray scattering (GIWAXS) reveals that PBFDO chains primarily adopt an edge‐on orientation with respect to the substrate in pristine films (Figure S10, Supporting Information), with a strong lamellar (100) peak at *Q*_z_ = 0.579 Å^−1^ (*d*‐spacing = 10.839 Å) and a π–π stacking (010) peak at *Q*_xy_  =  1.877 Å^−1^ (*d*‐spacing = 3.347 Å). This tightly packed microstructure is attributed to the absence of side chains and the high planarity of the PBFDO polymer backbone.^[^ ^21^ ^]^ Following PEIE treatment, the PBFDO/PEIE bilayer largely maintains the diffraction pattern of the original PBFDO. Note that the diffraction pattern of pure PEIE shows a broad ring, which is characteristic of a disordered system (Figure S10, Supporting Information).

**Figure 3:**
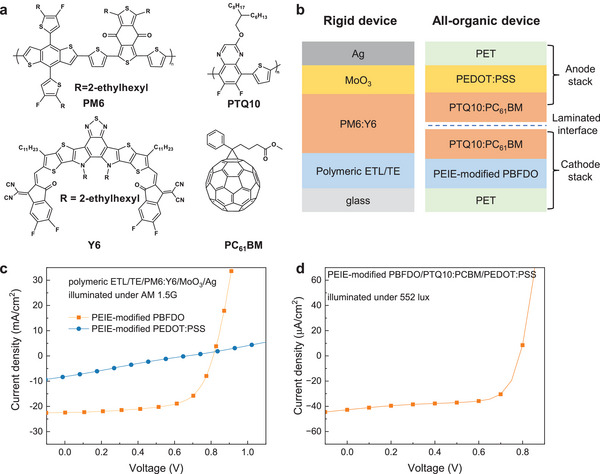
a) Chemical structures of the active layer materials and b) solar cell configurations used in this work. Current density‐voltage characteristics of c) rigid solar cells under AM 1.5G and d) all‐organic solar cells under 552 lux illumination.

After PEIE modification, PBFDO exhibits low sheet resistance and low absorption in the visible range, making it a potential alternative to ITO as a TE. The low WF of PEIE‐modified PBFDO also matches the lowest unoccupied molecular orbital of most common acceptors, allowing it to directly extract electrons from the active layer and function as a two‐in‐one ETL/TE.^[^ ^27^ ^]^ We then investigated its application as a two‐in‐one ETL/TE in OSCs with the active layer and device structures presented in **Figure** **3**a,b. Rigid solar cells with structure glass/PEIE‐modified PBFDO/PM6:Y6/MoO_3_/Ag were fabricated following the procedure outlined in the Experimental Section. The current density‐voltage (*J‐V*) characteristics, tested under 100 mW cm^−2^ AM 1.5G illumination and reported in Figure 3c, reveal a PCE of 12.1% (*V*_OC_ = 0.81 V, *J*_SC_ = 22.46 mA cm^−2^, and FF = 0.66). The performance of devices manufactured using different concentrations of PEIE is present in Figure S11, Supporting Information. Devices featuring lower concentrations of PEIE (i.e., 0.05, 0.10, and 0.20 wt%) showcase similar *J‐V* characteristics, with the 0.10 wt% PEIE modification exhibiting the highest efficiency among these concentrations. Conversely, an appreciable decrease in device performance is visible at a higher concentration of PEIE (0.50 wt%). Similar results are achieved when PBFDO is modified with sol‐gel amine‐based ZnO precursors, yielding OSCs with PCE of 13.35% (*V*_OC_ = 0.83 V, *J*_SC_ = 22.65 mA cm^−2^, and FF = 0.71, see Figure S12, Supporting Information). The *J*_SC_ was further confirmed by external quantum efficiency (EQE) measurements (Figure S13, Supporting Information) and optical modeling (Figure S14, Supporting Information). For comparison, devices featuring pure PBFDO (without PEIE) as the TE exhibit no photovoltaic behavior, a phenomenon we attributed to the relatively high WF of PBFDO itself (see Figure S15, Supporting Information). Additionally, we compared the device characteristics of identical OSCs comprising PEIE‐modified PEDOT:PSS as the TE and observed PCE < 2% (Figure 3c). We attributed this to the presence of a de‐doped PEIE‐modified PEDOT:PSS layer at the interface with the active layer. The *J‐V* characteristics of reference devices with ITO electrodes are presented in Figure S16, Supporting Information and show PCEs of about 13.99% (*V*_OC_ = 0.81 V, *J*_SC_ = 25.40 mA cm^−2^, and FF = 0.69) for PEIE‐modified ITO electrodes and 15.40% (*V*_OC_ = 0.83 V, *J*_SC_ = 25.42 mA cm^−2^, and FF = 0.73) for ZnO‐modified ITO electrodes. While the ITO‐based device shows a higher *J*_SC_ due to its relatively higher transmittance (Figure S17, Supporting Information), the similar *V*_OC_ and FF suggest that the sheet resistance of PEIE‐modified PBFDO electrode is sufficiently low to extract electrons from the active layer effectively.

This allows us to demonstrate flexible, all‐organic solar cells using the lamination approach described in the Experimental Section (Figure 3b). In brief, PEIE‐modified PBFDO was deposited on a PET foil and used as the low‐WF electrode, while PEDOT:PSS deposited on another PET foil served as the high‐WF electrode. A blend of PTQ10:PC_61_BM was utilized as the active layer and deposited on each PET sheet before the two were laminated on top of each other. PTQ10 and PC_61_BM were chosen as the active layer components for the all‐organic solar cells owing to their high stability^[^ ^28^ ^]^ and low synthetic complexity,^[^ ^29^ ^]^ rendering them conducive for large‐scale fabrication efforts. In addition, their absorbance spectra match that of indoor LED emission^[^ ^30^ ^]^ (Figure S18, Supporting Information). The resulting all‐organic solar cells exhibited a PCE of 15.1% (*V*_OC_ = 0.78 V, *J*_SC_ = 42.77 µA cm^−2^, and FF = 0.67) under 552 lux illumination conditions (Figure 3d). Remarkably, the all‐organic solar cells maintained nearly 90% of their initial efficiency even after 264 h of thermal aging at 70 °C and 94% of their initial efficiency after 192 h of continuous illumination under 1200 lux LED without encapsulation (Figure S19, Supporting Information). Additionally, these devices exhibited resilience to mechanical stress, enduring 1000 bending cycles to a bending radius of 4.0 mm without experiencing any loss in performance (Figure S20, Supporting Information). The device metrics are summarized in Table S1, Supporting Information, while the efficiency distribution is reported in Figure S21, Supporting Information. These figures of merit are among the highest reported for all‐organic solar cells tested under indoor lighting conditions (see Table S2, Supporting Information for a survey of the field).

## 3 Conclusions

In conclusion, we have demonstrated that treating the n‐type conducting polymer PBFDO with an ultrathin layer of PEIE produces a two‐in‐one electrode with a unique combination of low sheet resistance (130 Ω sq^−1^), low WF (4.2 eV), and high optical transparency (>85%) in the UV–vis‐NIR spectral region, outperforming conventional polymeric ETL materials (Table S3, Supporting Information).^[^ ^20^, ^31^ ^]^ Importantly, the doping level of PBFDO is not affected by PEIE treatment, ensuring stable and reliable performance. Utilizing the PEIE‐modified PBFDO electrode as a two‐in‐one ETL/TE in OSCs yields results comparable to those obtained with traditional ITO electrodes. Through a facile lamination approach, we have demonstrated the fabrication of all‐organic solar cells, achieving a record‐high power conversion efficiency (>15.1%) under indoor lighting conditions. This innovative approach offers a compelling alternative to traditional ITO electrodes in solar cells and holds promise for the development of efficient and cost‐effective optoelectronic devices.

## 4 Experimental Section

### Materials

PEDOT:PSS (Clevios PH 1000) was purchased from Heraeus. 5% ethylene glycol was added to PEDOT:PSS to improve its conductivity further. PBFDO was synthesized in dimethyl sulfoxide (DMSO) solution following a procedure reported previously.^[^ ^21^ ^]^ PEIE was purchased from Sigma‐Aldrich, and the active layer materials in OSCs were purchased from 1‐Material Inc.

### Thin Film Fabrication

Thin films of PBFDO were prepared by spin‐coating a DMSO‐based solution onto glass or PET substrates inside an N_2_‐filled glovebox, followed by annealing at 50 °C for 20 min and 150 °C for 10 min. PEDOT:PSS was spin‐coated onto a glass substrate in air and annealed at 150 °C for 10 min. The thickness of PBFDO and PEDOT:PSS films cast at 1000 rpm were ≈120 and 40 nm, respectively, as measured by stylus profilers (Dektak XT Bruker). The optical transmittance of PBFDO films as a function of film thickness is shown in Figure S22, Supporting Information. To modify their electronic properties, PEIE aqueous solution with different concentrations was spin‐coated on top of them at 5000 rpm and subsequently annealed at 150 °C for 10 min. The successful modification can be confirmed by the reduced contact angle, which decreased from ≈33° for PBFDO to 11° for PEIE‐modified PBFDO due to the hydrophilicity of PEIE (Figure S23, Supporting Information).

### UPS and XPS Spectroscopy

UPS and XPS spectra were acquired using a Scienta ESCA 200 system equipped with a SES 200 electron analyzer at 1 × 10^−10^ mbar. A monochromatic Al Ka X‐ray source (1486.6 eV) and a helium discharge lamp (21.22 eV) were used for XPS and UPS measurements. All spectra were collected at normal emission and were calibrated by a sputter‐cleaned Au film with the Fermi level at 0 eV and the Au 4f peak at 84.0 eV.

### Kelvin Probe

Kelvin probe measurements were performed using a scanning Kelvin Probe (SKP5050) in ambition conditions. The samples were spin‐coated on a clean glass substrate, and a highly ordered pyrolytic graphite with a WF of 4.6 eV was used for calibration. The statistical results are calculated from 30 measurements.

### Absorption Spectroscopy

The transmittance (*T*%) and reflectance (*R*%) spectra were measured with a UV–vis‐NIR spectrophotometer (PerkinElmer Lambda 950) with a step of 2 nm. The absorption (*A*%) spectra were calculated using the following equation: *A* = 100‐*T*‐*R*.

### EPR Spectra

Quantitative EPR measurements were performed with SPINSCAN X in dark conditions at room temperature. The PEDOT:PSS and PBFDO film were drop‐casted on a quartz substrate and then loaded into sealed quartz tubes.

### Electrical Characterization

Electrical conductivity measurements were performed using four probe techniques in ambient conditions. The *I–V* curves were recorded using a Keithley 4200‐SCS semiconductor characterization system.

### Thin‐Film Morphology Characterization

The AFM images were obtained using a silicon nitride cantilever with a spring constant of 40 N m^−1^ operated in tapping mode (Bruker Dimension Icon XR). The GIWAXS diffraction patterns were collected at a photon energy of 11.08 eV at an incidence angle of 0.12° at Beamline 9A in the Pohang Accelerator Laboratory (South Korea). All samples were characterized in vacuum, and the exposure time was 10 s.

### Thermal Analysis

Thermogravimetric analysis (TGA) was measured using a TA Instrument Q500 analyzer under N_2_ with a heating rate of 10 °C min^−1^. Differential scanning calorimetry (DSC) analysis was measured by TA Instruments Discovery DSC250 under N_2_. The samples were heated and cooled between 20 and 250 °C at a heating/cooling rate of 10 °C min^−1^. The materials were drop‐cast on the glass substrate and peeled off for measurements. PEIE‐modified PBFDO shows no sign of degradation under 200 °C (Figures S24 and S25, Supporting Information).

### Flexibility Test

The polymeric films spin‐coated on a PET foil and the all‐organic devices were placed on a cylinder with a radius of 2.5 mm or 4.0 mm and underwent repeated bending at a 180° angle for 1000 cycles to assess changes in morphology, conductivity, and photovoltaic performance. A schematic diagram of the bending test is shown in Figure S9b, Supporting Information.

### OSC Fabrication and Characterization

The rigid OSCs were fabricated using the configuration glass/PEIE‐modified polymeric TE/PM6:Y6/MoO_3_/Ag. The polymeric electrode was deposited on pre‐cleaned glass substrates via off‐center spin‐coating. The PEDOT:PSS and PBFDO electrodes were deposited as described above. The PEIE (0.1 wt% in H_2_O) solution was then spin‐coated on the polymeric electrode and annealed at 150 °C for 10 min. For the sol‐gel‐based device, the ZnO layer was deposited following previous reports.^[^ ^19^ ^]^ The active layer was then spin‐coated from PM6:Y6 solution (7:8.4 mg mL^−1^ in chloroform with 0.5 vol% of 1‐chloronaphthalene as additive) at 2500 r.p.m. and annealed at 100 °C for 10 min in a N_2_‐filled glovebox. Finally, MoO_3_ (10 nm)/Ag (100 nm) electrode was evaporated under a vacuum of 5 × 10^−7^ Torr. The effective area of the device is about 4.3 mm^2^, confirmed by a shadow mask.

The all‐organic devices were fabricated via solution processing and lamination technology following previous reports,^[^ ^30^ ^]^ except for the PEIE‐modified PBFDO as the cathode electrode, which was deposited as described above.

For the devices illuminated under AM 1.5G, the *J–V* curves were recorded by a Paios platform in the N_2_‐filled glovebox with a step voltage of 0.02 V. The light intensity (100 mW cm^−2^) was determined using a standard silicon photodiode. For indoor devices, the *J*‐*V* curves were acquired using a Keithley 2400 source meter while the devices were under cool LED irradiation at room temperature. The LED source's emission spectra and irradiance were measured using a high‐precision fiber optics spectrometer (QE‐Pro, Ocean Optics) and a Hamamatsu silicon photodiode (S1133‐01).^[^ ^32^ ^]^ By integrating the corresponding emission spectrum obtained from the specific device location, the illuminance, power density, and current density were calculated as 552 lux, 148 µW cm^−2^, and 67 µA cm^−2^, respectively. The EQE spectrum was recorded by a Newport Merlin lock‐in amplifier.

### Device Attenuation Test

For the thermal stability test, the devices were placed in a temperature‐controlled box set to 70 °C. For the photostability test, the devices were illuminated under 1200 lux. All tests were performed in an N_2_‐filled glovebox.

## Conflict of Interest

T.L., C.‐Y.Y., and S.F. filed provisional patent applications related to this work (application no. PCT/EP2023/063341). C.‐Y.Y., M.‐A.S., and S.F. are the co‐founders of n‐Ink AB. M.‐A.S. is an employee of n‐Ink AB. J.B. and T.Ö. are co‐founders and employees of Epishine AB. The other authors declare that they have no competing interests.

## Author Contributions

T.L. and G.B. contributed equally to this work. T.L. and S.F. conceived and designed the project. Q.L. and J.‐D.H. synthesized PBFDO. T.L. performed electrical measurements. Q.Z. and M.F. recorded and analyzed the UPS and XPS spectra. T.L. and Y.L. performed optical measurements. M.A.S. and M.X. recorded and analyzed the AFM. T.P.A.v.d.P. conducted the optical simulations. S.Y.J., C.‐Y.Y., and H.Y.W. measured and analyzed the GIWAXS data. T.L., Q.Z., Y.L., and F.G. fabricated and characterized the rigid solar cells. G.B., J.B., and T.Ö. fabricated and characterized the all‐organic solar cells. T.L. and S.F. wrote the manuscript. All authors contributed to the discussion and manuscript preparation.

## Supporting information

Supporting Information

## Data Availability

The data that support the findings of this study are available from the corresponding author upon reasonable request.
