# Supplementary material for: A Polymeric Two‐in‐One Electron Transport Layer and Transparent Electrode for Efficient Indoor All‐Organic Solar Cells
Source: Adv Sci (Weinh). 2024 Aug 29;11(40):2405676. doi: 10.1002/advs.202405676 (PMC11516159; doi:10.1002/advs.202405676)
Supplement: Supplementary file 1 — Supporting Information [file ADVS-11-2405676-s001.pdf]

ADVANCED SCIENCE
Open Access

Supporting Information

for *Adv. Sci.*, DOI 10.1002/advs.202405676

A Polymeric Two-in-One Electron Transport Layer and Transparent Electrode for Efficient Indoor All-Organic Solar Cells

*Tiefeng Liu, Gulzada Beket, Qifan Li, Qilun Zhang, Sang Young Jeong, Chi-Yuan Yang, Jun-Da Huang, Yuxuan Li, Marc-Antoine Stoeckel, Miao Xiong, Tom P. A. van der Pol, Jonas Bergqvist, Han Young Woo, Feng Gao, Mats Fahlman, Thomas Österberg and Simone Fabiano**

Supporting Information

A polymeric two-in-one electron transport layer and transparent electrode for efficient indoor all-organic solar cells

*Tiefeng Liu, Gulzada Beket, Qifan Li, Qilun Zhang, Sang Young Jeong, Chi-Yuan Yang, Jun-Da Huang, Yuxuan Li, Marc-Antoine Stoeckel, Miao Xiong, Tom P. A. van der Pol, Jonas Bergqvist, Han Young Woo, Feng Gao, Mats Fahlman, Thomas Österberg, Simone Fabiano**

T. Liu, Q. Li, Q. Zhang, C.-Y. Yang, J.-D. Huang, M.-A. Stoeckel, M. Xiong, T.P.A.v.d.Pol, M. Fahlman, S. Fabiano
Laboratory of Organic Electronics, Department of Science and Technology, Linköping University, SE-60174 Norrköping, Sweden
E-mail: simone.fabiano@liu.se

T. Liu, M.-A. Stoeckel, S. Fabiano
Wallenberg Initiative Materials Science for Sustainability, Department of Science and Technology, Linköping University, SE-60174 Norrköping, Sweden

G. Beket, Y. Li, F. Gao
Electronic and Photonic Materials, Department of Physics, Chemistry, and Biology, Linköping University, Linköping SE-58183, Sweden

G. Beket, J. Bergqvist, T. Österberg
Epishine AB, Attorpsgatan 2, SE-58273, Linköping, Sweden

Q. Zhang, J.-D. Huang, M. Fahlman, S. Fabiano
Wallenberg Wood Science Center, Department of Science and Technology (ITN), Linköping University, SE-60174 Norrköping, Sweden

S. Y. Jeong, H. Y. Woo
Department of Chemistry, College of Science, Korea University, 145 Anam-ro, Seongbuk-gu, Seoul 02841, Republic of Korea

C.-Y. Yang, M.-A. Stoeckel, S. Fabiano
n-Ink AB, Bredgatan 33, SE-60174 Norrköping, Sweden

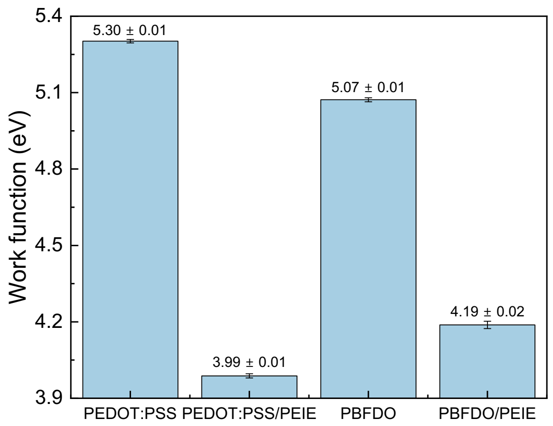

Figure S1. Work function of PEDOT:PSS and PBFDO films with and without PEIE surface modification as measured by Kelvin probe in ambient. The error bars indicate SD based on 30 measurements.

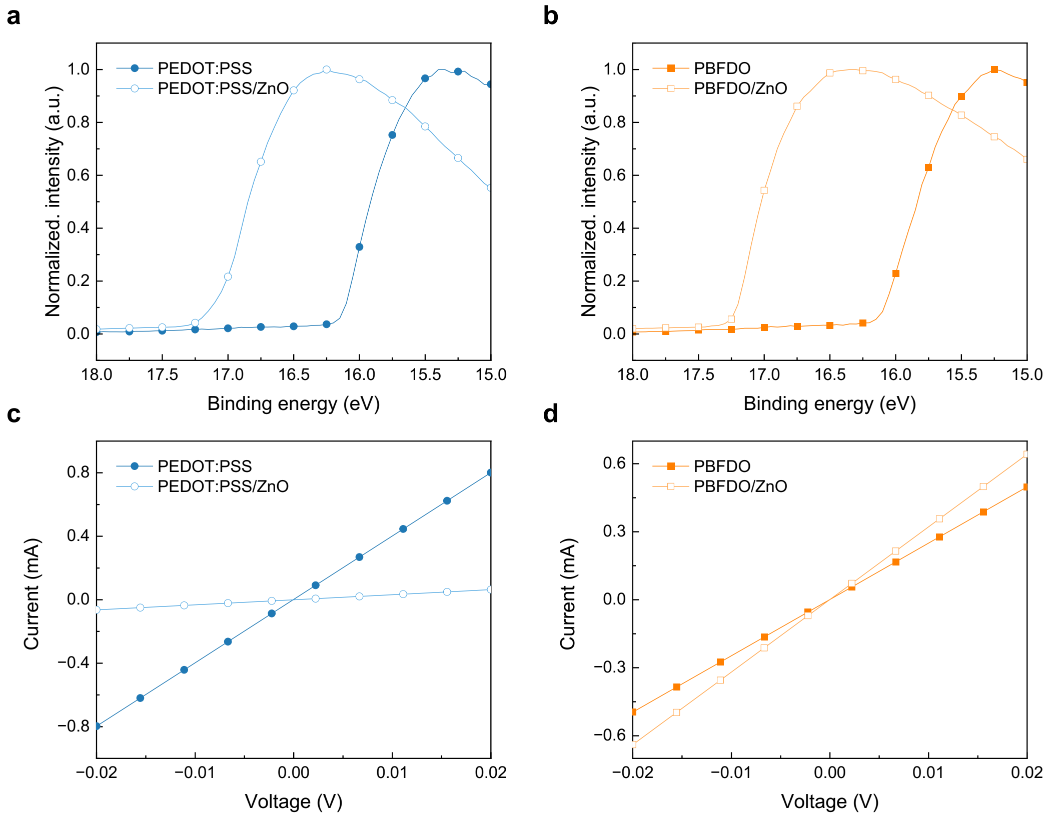

Figure S2. UPS spectra of (a) PEDOT:PSS and (b) PBFDO films before and after deposition of the ethanolamine-based ZnO precursor. Current-voltage curves of (c) PEDOT:PSS and (d) PBFDO films before and after deposition of the ethanolamine-based ZnO precursor.

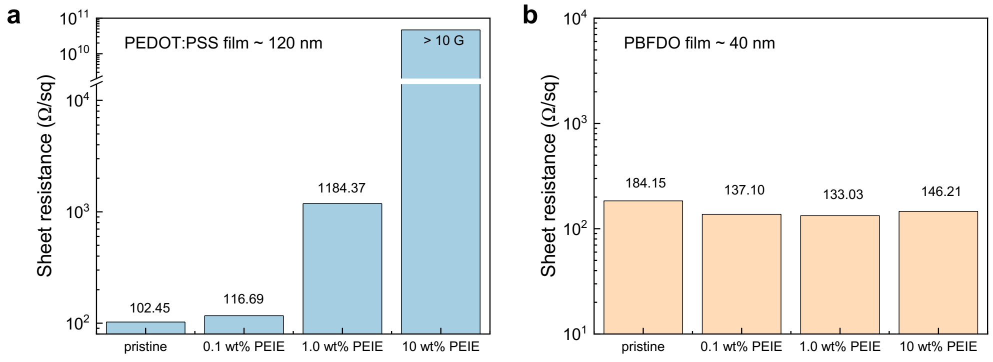

Figure S3. Sheet resistance of (a) PEDOT:PSS and (b) PBFDO films modified by PEIE (with different solution concentrations), as extracted from the current-voltage curves of Figure 1c-d.

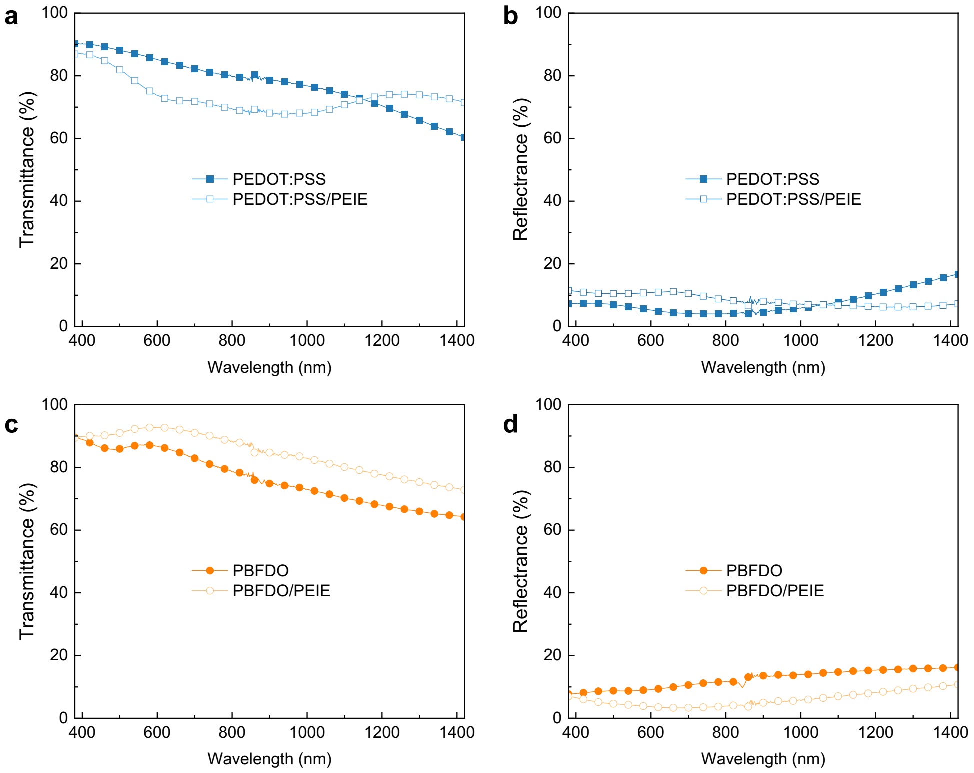

Figure S4. Transmittance and reflectance spectra of PEDOT:PSS (a-b) and PBFDO (c-d) films before and after modification with PEIE (1 wt%). Dedoping is observed for PEDOT:PSS films after PEIE modification: the transmittance decreases in the 400-1100 nm range and increases at wavelengths > 1100 nm. In stark contrast, the transmittance of PEIE-modified PBFDO films increases in the visible-infrared range, as shown in (c). We attributed this shift to the differences in the refractive indices of PBFDO (0.6 to 1.4[1]) and PEIE (about 1.6[2]). PEIE acts as an anti-reflective layer (reflectance spectra in d), having a similar effect to the commonly used dielectric-metal-dielectric transparent electrode.[3] The refractive index of PEDOT:PSS is about 1.5-1.6[4], on par with that of PEIE.

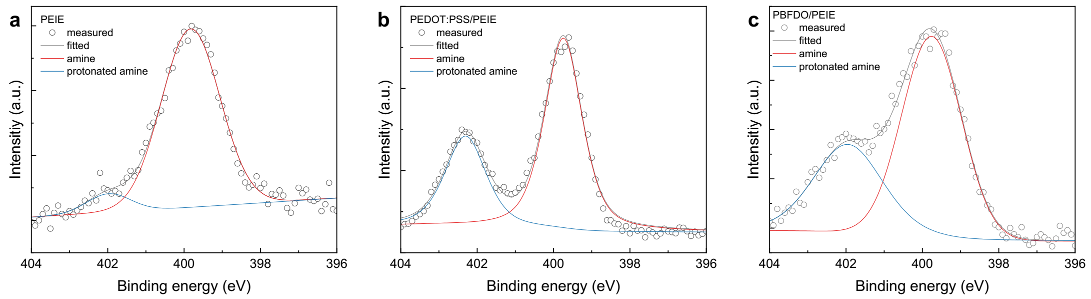

Figure S5. XPS N(1s) spectra of (a) PEIE, (b) PEIE-modified PEDOT:PSS, and (c) PEIE-modified PBFDO films. The protonated amine peak intensity increases after PEIE modification, indicating interaction with the conducting polymer.

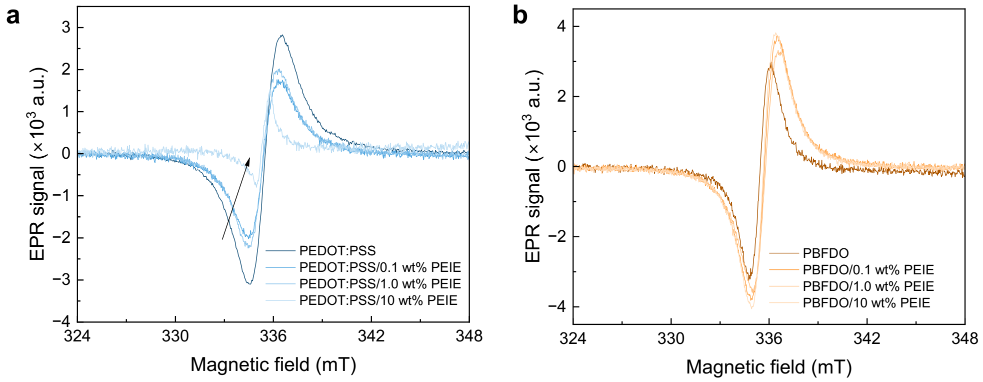

Figure S6. EPR spectra of (a) PEDOT:PSS and (b) PBFDO films at different concentrations of PEIE.

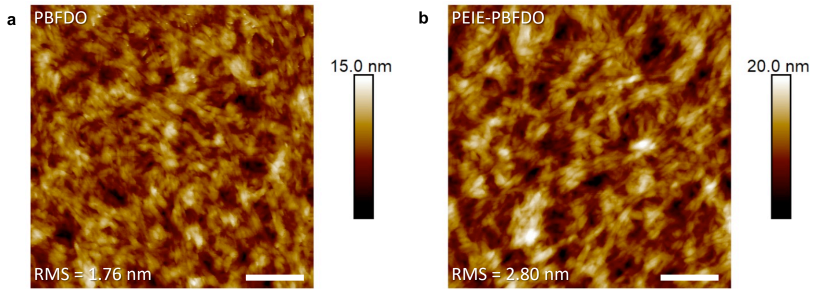

Figure S7. AFM images of (a) PBFDO and (b) PEIE-modified PBFDO films on glass substrate. The scale bar is 200 nm.

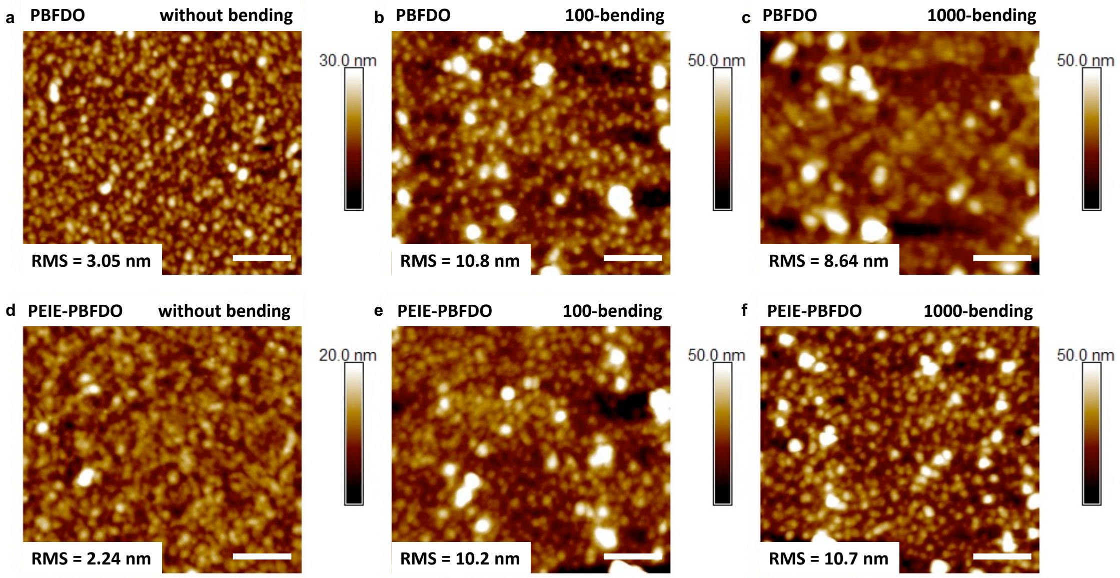

Figure S8. AFM images of PBFDO films on flexible PET substrate before bending (a), after 100 bending cycles (b), and after 1000 bending cycles (c). AFM images of PEIE-modified PBFDO films on flexible PET substrate before bending (d), after 100 bending cycles (e), and after 1000 bending cycles (f). The bending radius is 2.5 mm, and the bending angle is 180°. The scale bar is 400 nm.

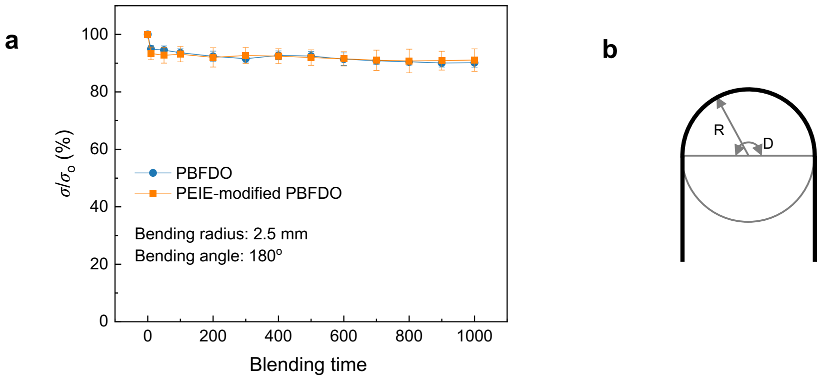

Figure S9. (a) Conductivity of PBFDO and PEIE-modified PBFDO films on a PET substrate after the bending test. (b) Schematic diagram of the bending test, where the bending radius (R) is 2.5 mm and the bending angle (D) is 180º.

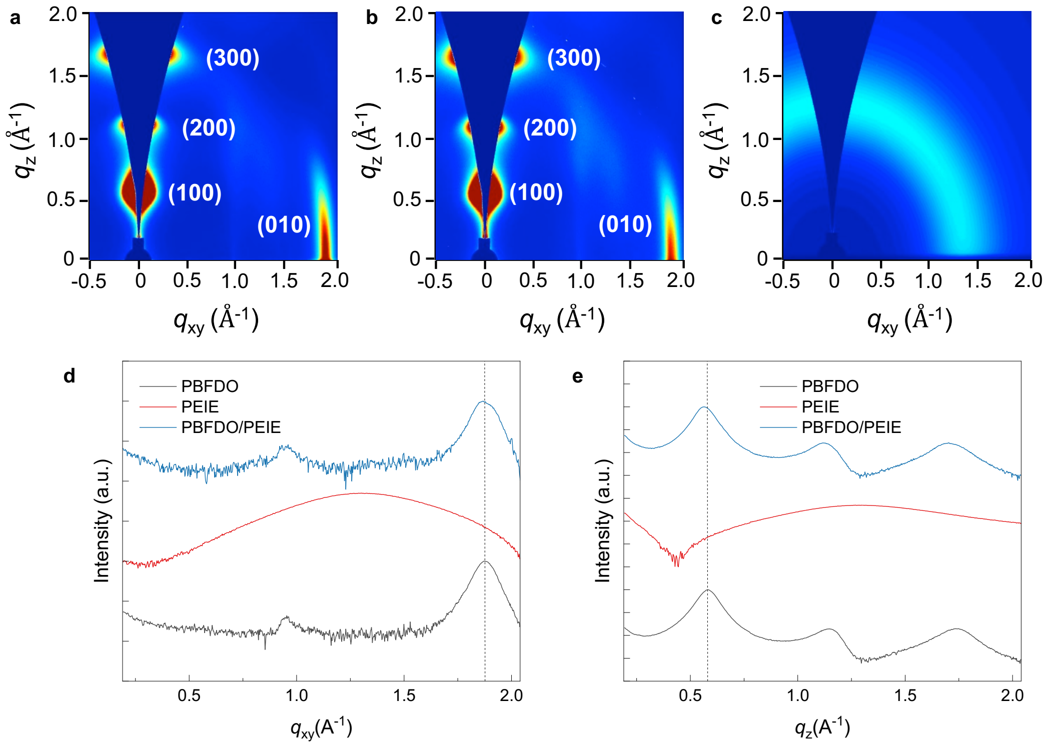

Figure S10. 2D GIWAXS patterns of (a) PBFDO, (b) PBFDO/PEIE, and (c) PEIE. In-plane (d) and out-of-plane (e) GIWAXS line cuts of PBFDO, PEIE, and PBFDO/PEIE films.

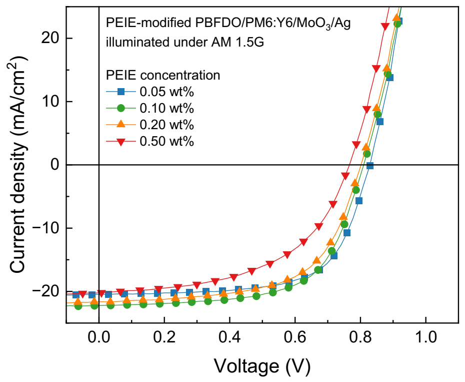

Figure S11. *J-V* curves of PEIE-modified PBFDO as a two-in-one electrode for different PEIE concentrations.

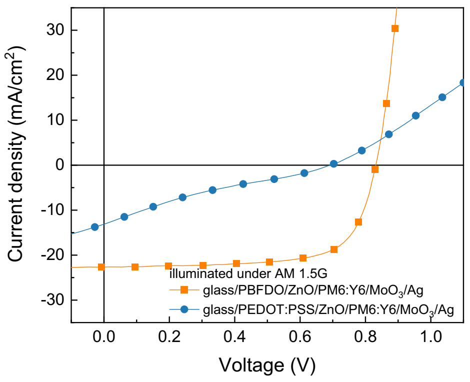

Figure S12. *J-V* characteristics of the rigid OSCs with polymeric TE and sol-gel ZnO as ETL under AM 1.5 G.

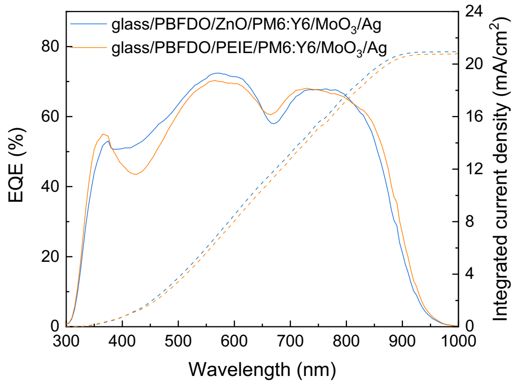

Figure S13. EQE spectra of OSCs with PBFDO as the transparent electrode. The integrated J_{SC} is consistent with the J_{SC} extracted from Figure 3c and Figure S12.

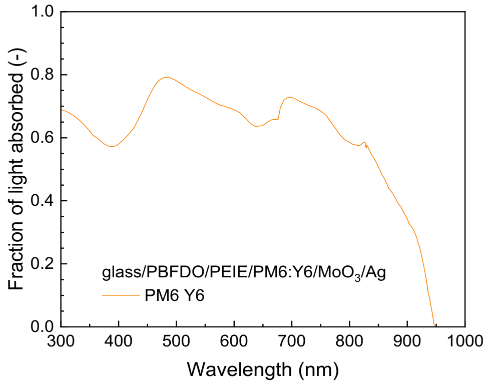

Figure S14. Fraction of light absorbed as calculated using the transfer matrix formalism incorporated in an in-house script based on the work of Burkhard et al.[5] The simulated device stack consisted of glass/PBFDO (50 nm)/PEIE (5 nm)/PM6:Y6 (100 nm)/MoO_3 (10 nm)/Ag (100 nm). The n and k values of the materials were obtained from the literature.[1-2, 6]

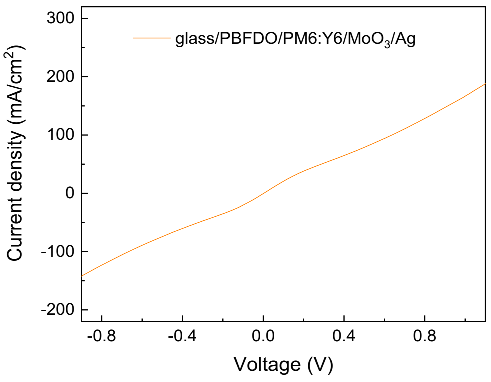

Figure S15. *J-V* curves of the device under AM 1.5G condition. The device structure is glass/PBFDO/PM6:Y6/MoO_3/Ag.

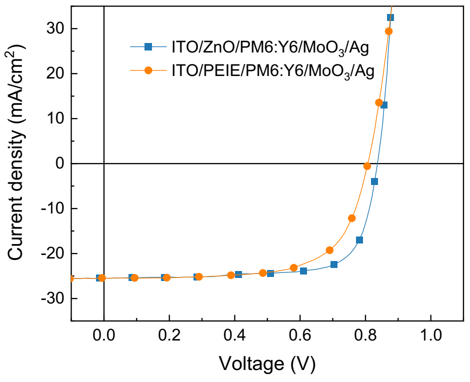

Figure S16. *J-V* curves of the reference device under AM 1.5G condition. The structure of the reference device is glass/ITO/PEIE or ZnO/PM6:Y6/MoO_3/Ag.

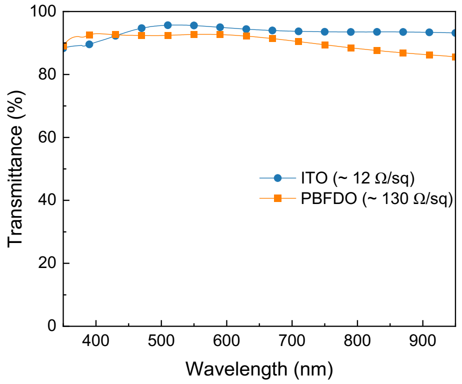

Figure S17. Transmittance of PEIE-modified ITO and PBFDO electrode.

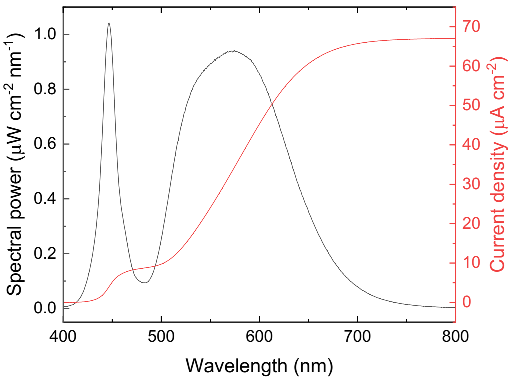

Figure S18. Photon flux (black line) and integrated current density (red line) of the LED lamp used in this work. The illuminance was obtained at about 552 lux by integrating spectral power.

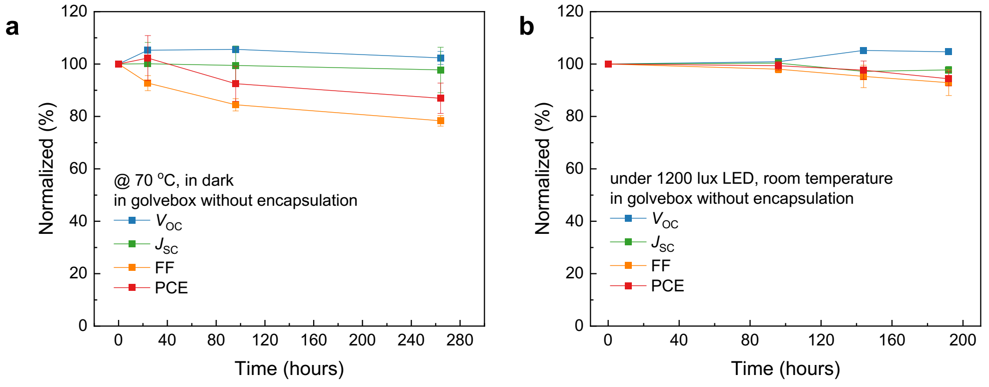

Figure S19. Stability tests of the all-organic solar cells. (a) Thermal aging at 70 °C in the dark. (b) Photostability under 1200 lux LED at room temperature. The devices were stored in an N_2-filled glovebox without encapsulation.

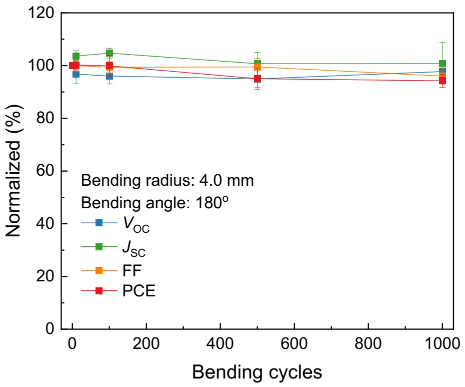

Figure S20. Mechanical stability of the all-organic solar cells (bending radius is 4.0 mm and the bending angle is 180°).

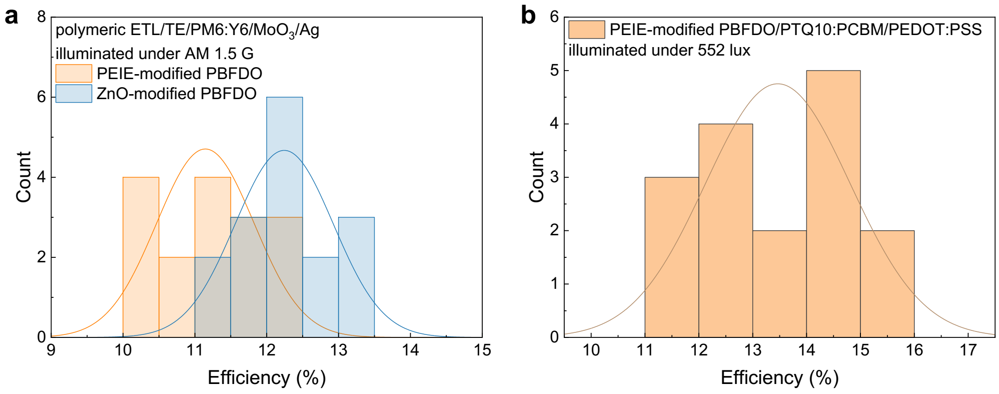

Figure S21. Efficiency histogram obtained from 16 devices. (a) Rigid solar cells tested under AM 1.5G. (b) All-organic solar cells tested under 552 lux.

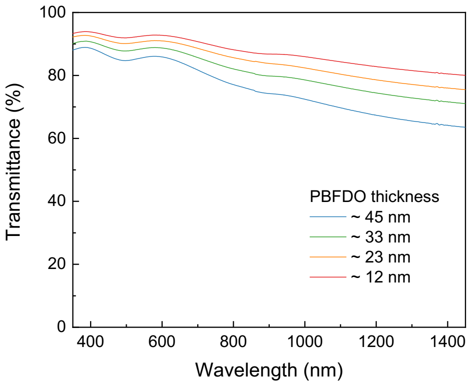

Figure S22. Transmittance of PBFDO films with different thicknesses.

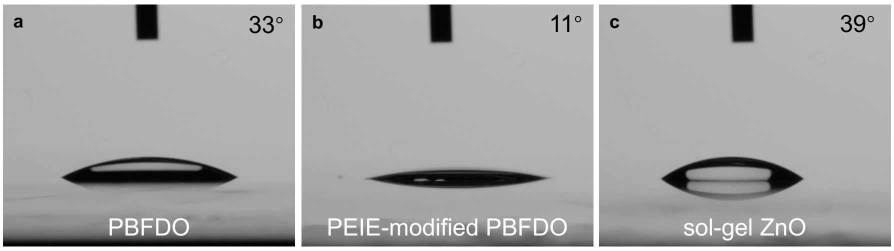

Figure S23. Contact angle of water droplets on (a) PBFDO and (b) PEIE-modified PBFDO films. The contact angle decreases from 33° for PBFDO to 11° for PEIE-modified PBFDO films, indicating the successful surface modification of PBFDO due to the hydrophilicity of PEIE. For comparison, the contact angle of water droplets on commonly used ZnO ETL (c) is about 39°.

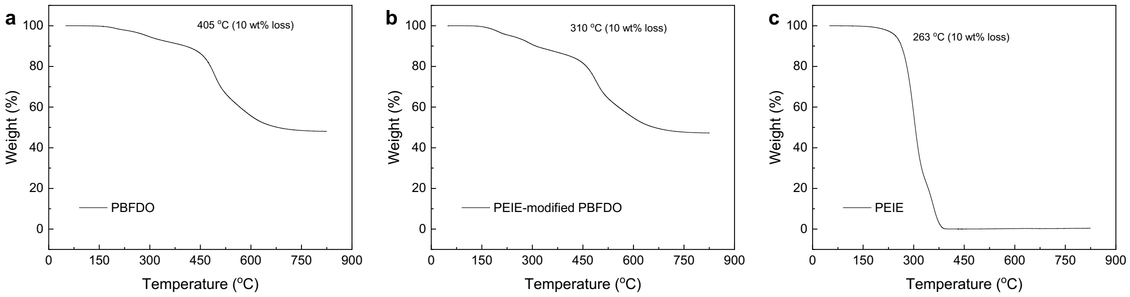

Figure S24. Thermogravimetric analysis (TGA) of (a) PBFDO, (b) PEIE-modified PBFDO, and (c) PEIE. Nearly no mass loss below 200 °C is observed for all the different samples. This temperature range is compatible with most flexible substrates (*e.g.*, 140 °C for PET and 170-180 °C for PEN)[7]. The heating rate is 10 °C min^{-1}.

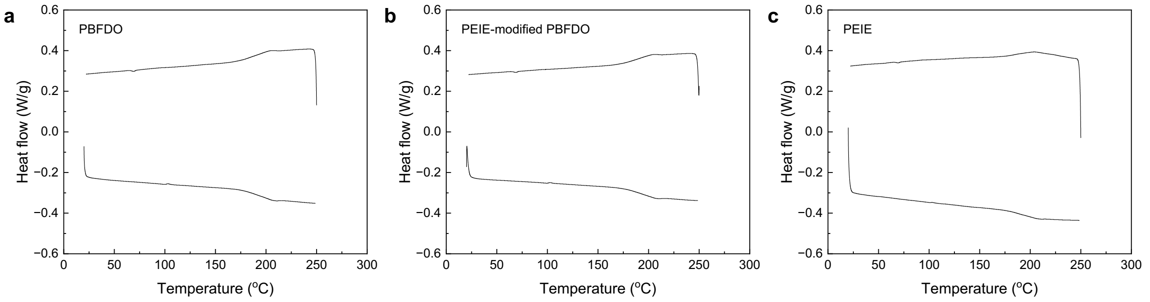

Figure S25. Differential scanning calorimetry (DSC) of (a) PBFDO, (b) PEIE-modified PBFDO, and (c) PEIE. No obvious exothermic or endothermic transformations are visible in the range of 20-250 °C at a heating/cooling rate of 10/10 °C min^{-1}.

Table S1. Photovoltaic parameters of the OSCs in this work. The devices were illuminated under AM 1.5G or 552 lux LED. The values in parentheses are the statistical results from 16 devices.

Structure	Illumination	V_{OC} (V)	J_{SC} (mA cm^{-2})	FF	PCE (%)
Glass/ITO/ZnO/PM6:Y6/MoO_3/Ag	100 mW cm^{-2}	0.83 (0.82±0.01)	25.42 (24.96±0.41)	0.73 (0.70±0.02)	15.40 (14.67±0.56)
Glass/PEIE-modified ITO/PM6:Y6/MoO_3/Ag		0.81 (0.80±0.01)	25.40 (24.67±0.45)	0.68 (0.66±0.02)	13.99 (13.23±0.63)
Glass/PBFDO/ZnO/PM6:Y6/MoO_3/Ag		0.83 (0.81±0.02)	22.65 (22.14±0.57)	0.71 (0.68±0.03)	13.35 (12.58±0.69)
Glass/PEIE-modified PBFDO/PM6:Y6/MoO_3/Ag		0.81 (0.79±0.02)	22.46 (21.78±0.65)	0.66 (0.61±0.04)	12.07 (11.21±0.83)
Glass/PEDOT:PSS/ZnO/PM6:Y6/MoO_3/Ag		0.69	13.33	0.21	1.93
Glass/PEIE-modified PEDOT:PSS/PM6:Y6/MoO_3/Ag		0.67	8.17	0.24	1.31
PET/PEIE-modified PBFDO/PTQ10:PC61BM/PEDOT:PSS/PET	552 lux	0.78 (0.77±0.05)	0.043 (0.041±0.005)	0.67 (0.60±0.10)	15.1 (13.8±1.13)

Table S2. Survey of all-organic solar cells.

**Structure**	**Substrate**	**Illumination**	**PCE (%)**	**Ref.**
PEIE-modified PBFDO/PTQ10:$PC_{61}BM$/PEDOT:PSS	PET	552 lux	15.1	This work
H_3PO_4–PEDOT:PSS/PEI/P3HT:ICBA/EG-PEDOT:PSS	PES	100 mW cm^{-2}	3.3	[8]
PEDOT:PSS/PEI/P3HT:ICBA/PEDOT:PSS		100 mW cm^{-2}	2.8	[9]
PH1000/PEI/P3HT:ICBA/PEDOT:PSS-m13/PEI/PTB7-Th:$PC_{61}BM$/PEDOT:PSS	PES	100 mW cm^{-2}	6.1	[10]
PEDOT:PSS/P3HT:ICBA:PEI/PEDOT:PSS	PES	100 mW cm^{-2}	3.3	[11]
HC-PEDOT:PSS/PEI/P3HT:ICBA/EG-PEDOT:PSS	PES	100 mW cm^{-2}	4.05	[12]
PH1000/PEI/P3HT:ICBA/PEDOT	PES	100 mW cm^{-2}	3.0	[13]
PH1000/PEIE/P3HT:IDT-2BR	PES	100 mW cm^{-2}	2.88	[14]
PEDOT:PSS/PEIx/TQ1:$PC_{61}BM$/PEDOT:PSS	PET	100 mW cm^{-2}	1.70	[15]
low-WF PEDOT:PSS/P3HT:ICBA/high-WF PEDOT:PSS	Glass	100 mW cm^{-2}	4.0	[16]
low-WF PEDOT:PSS/P3HT:ICBA/high-WF PEDOT:PSS	Glass	100 mW cm^{-2}	3.1	[17]
hc-PEDOT:PSS-TBA/PM6:IT-4F/PEDOT:PSS	Glass	100 mW cm^{-2}	7.82	[18]
H_2SO_4/EG-PEDOT:PSS/PM6:Y6/PDINO/PEDOT:PSS	Glass	100 mW cm^{-2}	8.93	[19]

Table S3. Survey of work function, conductivity, and transparency of conducting polymers.

Materials	Type	Conductivity (S/cm)	Work function (eV)	Transparency[a] @ 550 nm (%)	ref
$HClO_4$ treated PEDOT:PSS	P	4250	5.39	90.3	[20]
e-PEDOT:PSS	P	529	5.3	84.3	[21]
H_2SO_4 treated PEDOT:PSS	P	3210	5.1	~90	[22]
IL-PEDOT:PSS	P	1810	5.1	85.7	[23]
H_2SO_4/EG treated PEDOT:PSS	P	4200	4.87	73	[19]
MSA treated PEDOT:PSS	P	2540	4.8	~93	[24]
HQ treated PEDOT:PSS	P	1394	4.89	95.9	[25]
Na-MeSal treated PEDOT:PSS	P	584.2	4.7	86.5	[26]
PEDOT:PSS:SCNT	P	3500	4.4	~83	[27]
PEDOT:PSS-TBA	P	300	4.2	84	[18]
PEIE modified PEDOT	P	807	4.08	~90	[28]
PEDOT:FTS	P	142	4.37	81	[29]
VPP-PEDOT	P	385	4.3	N.A.	[30]
TDAE-PEDOT:TOS	P	1000	3.8	N.A.	[31]
PEIE modified PEDOT	P	1140	4	83	[16]
Ω-PEDOT	P	290	5	N.A.	[32]
PEDOT:PSS:CFE	P	> 4000	N.A.	> 80	[33]
PEDOT/STEC	P	3100	N.A.	96	[34]
PEDOT:PSS/PR	P	700	4.9	92	[35]
PEDOT:PSS with ION E and PEIE	P	~1000	~4.8	N.A.	[36]
BBL:PEI	N	8	4.19	N.A.	[37]
BBL:PCAT-K	N	2.3	3.93	N.A.	[38]
NHC doped FBDPPV	N	4.07	8.4	N.A.	[39]
N-DMBI doped FBDPPV	N	4.19	14	N.A.	[40]
TAM doped FBDPPV-OEG	N	39	N.A.	N.A.	[41]
TAM-doped FBDPPV	N	21	N.A.	N.A.	[42]
N-DMBI-H doped TBDPPV	N	90	3.60	N.A.	[43]
N-DMBI-H doped TBDPPV-T	N	59	3.86	N.A.	[43]
TAM doped UFBDPPV	N	22	4.13	N.A.	[44]
N-DMBI-H doped UFBDPPV	N	16	3.90	N.A.	[44]
N-DMBI catalytic-doped f-BTI2TEG-FT	N	104	N.A.	N.A.	[45]
N-DMBI doped f-BSeI2TEG-FT	N	103.5	N.A.	N.A.	[46]
N-DMBI doped PDTzTI-TEG	N	34	N.A.	N.A.	[47]
N-DMBI doped PDTzSI-Se	N	164.0	N.A.	N.A.	[48]
N-DMBI doped PDTzSI-T	N	62.0	N.A.	N.A.	[48]
N-DMBI doped PDTzTI-Se	N	98.0	N.A.	N.A.	[48]
N-DMBI doped PDTzTI-T	N	73.9	N.A.	N.A.	[48]
TDAE DOPED P(NDI2OD-Tz2)	N	0.1	~4.0	N.A.	[49]

n-DMBI doped TEG-N2200	N	0.17	~4.0	N.A.	[50]
N-DMBI doped PCNI2-BTI	N	150.2	N.A.	N.A.	[51]
N-DMBI doped PCNI-BTI	N	23.3	N.A.	N.A.	[52]
N-DMBI doped PClClTVT	N	38.3	N.A.	N.A.	[53]
N-DMBI doped PFClTVT	N	12.4	N.A.	N.A.	[53]
PSpF blend PDPIN	N	78.1	4.16	N.A.	[54]
PSpF blend PFClTVT	N	58	3.22	N.A.	[55]
n-PBDO	N	4.6	2100	80	[1]
PBFDO	N	4.67	2000	N.A.	[56]
**PEIE modified PBFDO**	**N**	**4.2**	**1824**	**92**	**This work**

[a]Transparency of films with a reasonable sheet resistance (~100 Ω/sq).

Reference.

[1] Z. Ke, A. Abtahi, J. Hwang, K. Chen, J. Chaudhary, I. Song, K. Perera, L. You, K. N. Baustert, K. R. Graham, J. Mei, *Journal of the American Chemical Society* **2023**, 145, 3706.

[2] S. Kim, A. Prasetio, J. W. Han, Y. Kim, M. Shin, J. Heo, J. H. Kim, S. Cho, Y. H. Kim, M. Jahandar, D. C. Lim, *Communications Materials* **2021**, 2, 26.

[3] a)C. Ji, D. Liu, C. Zhang, L. Jay Guo, *Nature Communications* **2020**, 11, 3367; b)C. Zhang, C. Ji, Y.-B. Park, L. J. Guo, *Advanced Optical Materials* **2021**, 9, 2001298; c)P. Kumar, S. You, A. Vomiero, *Advanced Energy Materials* **2023**, 13, 2301555.

[4] A. Elschner, S. Kirchmeyer, W. Lövenich, U. Merker, K. Reuter, *PEDOT Principles and Applications of an Intrinsically Conductive Polymer*, CRC press, **2010**.

[5] G. F. Burkhard, E. T. Hoke, M. D. McGehee, *Advanced Materials* **2010**, 22, 3293.

[6] a)W. Li, S. Zeiske, O. J. Sandberg, D. B. Riley, P. Meredith, A. Armin, *Energy & Environmental Science* **2021**, 14, 6484; b)S. Xie, R. Xia, Z. Chen, J. Tian, L. Yan, M. Ren, Z. Li, G. Zhang, Q. Xue, H.-L. Yip, Y. Cao, *Nano Energy* **2020**, 78, 105238.

[7] R. Po, A. Bernardi, A. Calabrese, C. Carbonera, G. Corso, A. Pellegrino, *Energy & Environmental Science* **2014**, 7, 925.

[8] W. Meng, R. Ge, Z. Li, J. Tong, T. Liu, Q. Zhao, S. Xiong, F. Jiang, L. Mao, Y. Zhou, *ACS Applied Materials & Interfaces* **2015**, 7, 14089.

[9] Y. Zhou, T. M. Khan, J. W. Shim, A. Dindar, C. Fuentes-Hernandez, B. Kippelen, *Journal of Materials Chemistry A* **2014**, 2, 3492.

[10] J. Tong, S. Xiong, Y. Zhou, L. Mao, X. Min, Z. Li, F. Jiang, W. Meng, F. Qin, T. Liu, R. Ge, C. Fuentes-Hernandez, B. Kippelen, Y. Zhou, *Materials Horizons* **2016**, 3, 452.

[11] L. Mao, B. Luo, L. Sun, S. Xiong, J. Fan, F. Qin, L. Hu, Y. Jiang, Z. Li, Y. Zhou, *Materials Horizons* **2018**, 5, 123.

[12] S. Nie, F. Qin, Y. Liu, C. Qiu, Y. Jin, H. Wang, L. Liu, L. Hu, Z. Su, J. Song, X. Yin, Z. Xu, Y. Yao, H. Wang, Y. Zhou, Z. Li, *Molecules* **2023**, 28, 2836.

[13] Y. Zhou, C. Fuentes-Hernandez, J. Shim, J. Meyer, A. J. Giordano, H. Li, P. Winget, T. Papadopoulos, H. Cheun, J. Kim, M. Fenoll, A. Dindar, W. Haske, E. Najafabadi, T. M. Khan, H. Sojoudi, S. Barlow, S. Graham, J.-L. Brédas, S. R. Marder, A. Kahn, B. Kippelen, *Science* **2012**, 336, 327.

[14] Y. Wang, B. Jia, F. Qin, Y. Wu, W. Meng, S. Dai, Y. Zhou, X. Zhan, *Polymer* **2016**, 107, 108.

[15] W. Cai, T. Österberg, M. J. Jafari, C. Musumeci, C. Wang, G. Zuo, X. Yin, X. Luo, J. Johansson, M. Kemerink, L. Ouyang, T. Ederth, O. Inganäs, *Journal of Materials Chemistry C* **2020**, 8, 328.

[16] Z. Li, Y. Liang, Z. Zhong, J. Qian, G. Liang, K. Zhao, H. Shi, S. Zhong, Y. Yin, W. Tian, *Synthetic Metals* **2015**, 210, 363.

[17] Z. F. Li, F. Qin, T. F. Liu, R. Ge, W. Meng, J. H. Tong, S. X. Xiong, Y. H. Zhou, *Organic Electronics* **2015**, 21, 144.

[18] T. Liu, L. Sun, X. Dong, Y. Jiang, W. Wang, C. Xie, W. Zeng, Y. Liu, F. Qin, L. Hu, Y. Zhou, *Advanced Functional Materials* **2021**, 31, 2107250.

[19] X. Fan, R. Wen, Y. Xia, J. Wang, X. Liu, H. Huang, Y. Li, W. Zhu, Y. Cheng, L. Ma, J. Fang, H. Tsai, W. Nie, *Solar RRL* **2020**, 4, 1900543.

[20] J. Wan, X. Fan, H. Huang, J. Wang, Z. Zhang, J. Fang, F. Yan, *Journal of Materials Chemistry A* **2020**, 8, 21007.

[21] X. Dong, X. Zhou, Y. Liu, S. Xiong, J. Cheng, Y. Jiang, Y. Zhou, *Energy & Environmental Science* **2023**, 16, 1511.

[22] N. Kim, H. Kang, J.-H. Lee, S. Kee, S. H. Lee, K. Lee, *Advanced Materials* **2015**, 27, 2317.

[23] H. Park, J.-H. Lee, S. Lee, S. Y. Jeong, J. W. Choi, C.-L. Lee, J.-H. Kim, K. Lee, *ACS Applied Materials & Interfaces* **2020**, 12, 2276.

[24] X. Fan, B. Xu, S. Liu, C. Cui, J. Wang, F. Yan, *ACS Applied Materials & Interfaces* **2016**, 8, 14029.

[25] I. Song, N. Yeon Park, G. Seung Jeong, J. Hwan Kang, J. Hwa Seo, J.-Y. Choi, *Applied Surface Science* **2020**, 529, 147176.

[26] L. Liu, L. Wu, H. Yang, H. Ge, J. Xie, K. Cao, G. Cheng, S. Chen, *ACS Applied Materials & Interfaces* **2022**, 14, 1615.

[27] X. Hu, L. Chen, L. Tan, T. Ji, Y. Zhang, L. Zhang, D. Zhang, Y. Chen, *Journal of Materials Chemistry A* **2016**, 4, 6645.

[28] Z. Li, F. Qin, T. Liu, R. Ge, W. Meng, J. Tong, S. Xiong, Y. Zhou, *Organic Electronics* **2015**, 21, 144.

[29] M. Kim, Y. S. Lee, Y. C. Kim, M. S. Choi, J. Y. Lee, *Synthetic Metals* **2011**, 161, 2318.

[30] A. Gadisa, K. Tvingstedt, S. Admassie, L. Lindell, X. Crispin, M. R. Andersson, W. R. Salaneck, O. Inganäs, *Synthetic Metals* **2006**, 156, 1102.

[31] L. Lindell, A. Burquel, F. L. E. Jakobsson, V. Lemaur, M. Berggren, R. Lazzaroni, J. Cornil, W. R. Salaneck, X. Crispin, *Chemistry of Materials* **2006**, 18, 4246.

[32] D. Corzo, E. Bihar, E. B. Alexandre, D. Rosas-Villalva, D. Baran, *Advanced Functional Materials* **2021**, 31, 2005763.

[33] X. Hu, X. Meng, L. Zhang, Y. Zhang, Z. Cai, Z. Huang, M. Su, Y. Wang, M. Li, F. Li, X. Yao, F. Wang, W. Ma, Y. Chen, Y. Song, *Joule* **2019**, 3, 2205.

[34] Y. Wang, C. Zhu, R. Pfattner, H. Yan, L. Jin, S. Chen, F. Molina-Lopez, F. Lissel, J. Liu, N. I. Rabiah, Z. Chen, J. W. Chung, C. Linder, M. F. Toney, B. Murmann, Z. Bao, *Science Advances* **2017**, 3, e1602076.

[35] Z. Zhang, W. Wang, Y. Jiang, Y.-X. Wang, Y. Wu, J.-C. Lai, S. Niu, C. Xu, C.-C. Shih, C. Wang, H. Yan, L. Galuska, N. Prine, H.-C. Wu, D. Zhong, G. Chen, N. Matsuhisa, Y. Zheng, Z. Yu, Y. Wang, R. Dauskardt, X. Gu, J. B. H. Tok, Z. Bao, *Nature* **2022**, 603, 624.

[36] N. Matsuhisa, S. Niu, S. J. K. O'Neill, J. Kang, Y. Ochiai, T. Katsumata, H.-C. Wu, M. Ashizawa, G.-J. N. Wang, D. Zhong, X. Wang, X. Gong, R. Ning, H. Gong, I. You, Y. Zheng, Z. Zhang, J. B. H. Tok, X. Chen, Z. Bao, *Nature* **2021**, 600, 246.

[37] C.-Y. Yang, M.-A. Stoeckel, T.-P. Ruoko, H.-Y. Wu, X. Liu, N. B. Kolhe, Z. Wu, Y. Puttisong, C. Musumeci, M. Massetti, H. Sun, K. Xu, D. Tu, W. M. Chen, H. Y. Woo, M. Fahlman, S. A. Jenekhe, M. Berggren, S. Fabiano, *Nature Communications* **2021**, 12, 2354.

[38] T. Liu, J. Heimonen, Q. Zhang, C.-Y. Yang, J.-D. Huang, H.-Y. Wu, M.-A. Stoeckel, T. P. A. van der Pol, Y. Li, S. Y. Jeong, A. Marks, X.-Y. Wang, Y. Puttisong, A. Y. Shimolo, X. Liu, S. Zhang, Q. Li,

M. Massetti, W. M. Chen, H. Y. Woo, J. Pei, I. McCulloch, F. Gao, M. Fahlman, R. Kroon, S. Fabiano, *Nature Communications* **2023**, 14, 8454.

[39] Y.-F. Ding, C.-Y. Yang, C.-X. Huang, Y. Lu, Z.-F. Yao, C.-K. Pan, J.-Y. Wang, J. Pei, *Angewandte Chemie International Edition* **2021**, 60, 5816.

[40] K. Shi, F. Zhang, C.-A. Di, T.-W. Yan, Y. Zou, X. Zhou, D. Zhu, J.-Y. Wang, J. Pei, *Journal of the American Chemical Society* **2015**, 137, 6979.

[41] X.-Y. Wang, Z.-D. Yu, Y. Lu, Z.-F. Yao, Y.-Y. Zhou, C.-K. Pan, Y. Liu, Z.-Y. Wang, Y.-F. Ding, J.-Y. Wang, J. Pei, *Advanced Materials* **2023**, 35, 2300634.

[42] C.-Y. Yang, Y.-F. Ding, D. Huang, J. Wang, Z.-F. Yao, C.-X. Huang, Y. Lu, H.-I. Un, F.-D. Zhuang, J.-H. Dou, C.-a. Di, D. Zhu, J.-Y. Wang, T. Lei, J. Pei, *Nature Communications* **2020**, 11, 3292.

[43] Y. Lu, Z.-D. Yu, H.-I. Un, Z.-F. Yao, H.-Y. You, W. Jin, L. Li, Z.-Y. Wang, B.-W. Dong, S. Barlow, E. Longhi, C.-a. Di, D. Zhu, J.-Y. Wang, C. Silva, S. R. Marder, J. Pei, *Advanced Materials* **2021**, 33, 2005946.

[44] Y. Lu, Z.-D. Yu, Y. Liu, Y.-F. Ding, C.-Y. Yang, Z.-F. Yao, Z.-Y. Wang, H.-Y. You, X.-F. Cheng, B. Tang, J.-Y. Wang, J. Pei, *Journal of the American Chemical Society* **2020**, 142, 15340.

[45] H. Guo, C.-Y. Yang, X. Zhang, A. Motta, K. Feng, Y. Xia, Y. Shi, Z. Wu, K. Yang, J. Chen, Q. Liao, Y. Tang, H. Sun, H. Y. Woo, S. Fabiano, A. Facchetti, X. Guo, *Nature* **2021**, 599, 67.

[46] J. Li, M. Liu, K. Yang, Y. Wang, J. Wang, Z. Chen, K. Feng, D. Wang, J. Zhang, Y. Li, H. Guo, Z. Wei, X. Guo, *Advanced Functional Materials* **2023**, 33, 2213911.

[47] Y. Shi, J. Li, H. Sun, Y. Li, Y. Wang, Z. Wu, S. Y. Jeong, H. Y. Woo, S. Fabiano, X. Guo, *Angewandte Chemie International Edition* **2022**, 61, e202214192.

[48] Y. Li, W. Wu, Y. Wang, E. Huang, S. Y. Jeong, H. Y. Woo, X. Guo, K. Feng, *Angewandte Chemie International Edition* **2024**, 63, e202316214.

[49] S. Wang, H. Sun, T. Erdmann, G. Wang, D. Fazzi, U. Lappan, Y. Puttisong, Z. Chen, M. Berggren, X. Crispin, A. Kiriy, B. Voit, T. J. Marks, S. Fabiano, A. Facchetti, *Advanced Materials* **2018**, 30, 1801898.

[50] J. Liu, L. Qiu, R. Alessandri, X. Qiu, G. Portale, J. Dong, W. Talsma, G. Ye, A. A. Sengrian, P. C. T. Souza, M. A. Loi, R. C. Chiechi, S. J. Marrink, J. C. Hummelen, L. J. A. Koster, *Advanced Materials* **2018**, 30, 1704630.

[51] K. Feng, W. Yang, S. Y. Jeong, S. Ma, Y. Li, J. Wang, Y. Wang, H. Y. Woo, P. K. L. Chan, G. Wang, X. Guo, M. Zhu, *Advanced Materials* **2023**, 35, 2210847.

[52] K. Feng, H. Guo, J. Wang, Y. Shi, Z. Wu, M. Su, X. Zhang, J. H. Son, H. Y. Woo, X. Guo, *Journal of the American Chemical Society* **2021**, 143, 1539.

[53] J. Han, H. Fan, Q. Zhang, Q. Hu, T. P. Russell, H. E. Katz, *Advanced Functional Materials* **2021**, 31, 2005901.

[54] J. Han, Y. Jiang, E. Tiernan, C. Ganley, Y. Song, T. Lee, A. Chiu, P. McGuiggan, N. Adams, P. Clancy, T. P. Russell, P. E. Hopkins, S. M. Thon, J. D. Tovar, H. E. Katz, *Angewandte Chemie International Edition* **2023**, 62, e202219313.

[55] J. Han, E. Tiernan, T. Lee, A. Chiu, P. McGuiggan, N. Adams, J. A. Tomko, P. E. Hopkins, S. M. Thon, J. D. Tovar, H. E. Katz, *Advanced Materials* **2022**, 34, 2201062.

[56] H. Tang, Y. Liang, C. Liu, Z. Hu, Y. Deng, H. Guo, Z. Yu, A. Song, H. Zhao, D. Zhao, Y. Zhang, X. Guo, J. Pei, Y. Ma, Y. Cao, F. Huang, *Nature* **2022**, 611, 271.
